# The 10,000-Year Success Story of Wheat!

**DOI:** 10.3390/foods10092124

**Published:** 2021-09-08

**Authors:** Telma de Sousa, Miguel Ribeiro, Carolina Sabença, Gilberto Igrejas

**Affiliations:** 1 Department of Genetics and Biotechnology, University of Trás-os-Montes and Alto Douro, 5000-801 Vila Real, Portugal; telmaslsousa@hotmail.com (T.d.S.); jmribeiro@utad.pt (M.R.); anacarolina@utad.pt (C.S.); 2 Functional Genomics and Proteomics Unity, University of Trás-os-Montes and Alto Douro, 5000-801 Vila Real, Portugal; 3 LAQV-REQUIMTE, Faculty of Science and Technology, University Nova of Lisbon, 2825-149 Lisbon, Caparica, Portugal

**Keywords:** wheat, *Triticum aestivum* L., *Triticum durum* Desf., gluten, breadmaking, celiac disease

## Abstract

Wheat is one of the most important cereal crops in the world as it is used in the production of a diverse range of traditional and modern processed foods. The ancient varieties einkorn, emmer, and spelt not only played an important role as a source of food but became the ancestors of the modern varieties currently grown worldwide. Hexaploid wheat (*Triticum aestivum* L.) and tetraploid wheat (*Triticum durum* Desf.) now account for around 95% and 5% of the world production, respectively. The success of this cereal is inextricably associated with the capacity of its grain proteins, the gluten, to form a viscoelastic dough that allows the transformation of wheat flour into a wide variety of staple forms of food in the human diet. This review aims to give a holistic view of the temporal and proteogenomic evolution of wheat from its domestication to the massively produced high-yield crop of our day.

## 1. Introduction

Wheat is a high-yielding crop that is easy to store and is very adaptable to different climates. From the most primitive form of wheat to the species currently grown, these and other desirable characteristics have been selected and developed by human societies since ancient times [1,2]. Its domestication is thought to have occurred in the Fertile Crescent about 10,000 years ago and has spread to all parts of the world through the first farmers, adapting the domesticated populations to different environments [3]. Wheat is able to grow in temperate, Mediterranean, and subtropical regions of the two hemispheres, mainly due to its enormous genetic diversity. For example, there are more than 25,000 varieties of *Triticum aestivum* L. adapted to different temperate environments [4]. Wheat can be classified according to when it is sown. Winter wheat is sown in autumn because the seedlings need a period during the vegetative phase when temperatures are between 0 and 5 °C. About 80% of the world’s wheat is winter wheat. Spring wheat is planted in spring and harvested in late summer or fall in South Asian countries or North Africa [5].

The most important varieties of modern wheat are tetraploid durum wheat (*Triticum durum*) and hexaploid bread wheat (*Triticum aestivum* L.), which have different attributes in terms of genomic composition, grain composition, and end use [6]. The progressive domestication of these wheat species led to the restructuring of the rachis and glumes, converting the brittle ears of wild species into non-brittle and bare grain ears in cultivated species [7]. These morphological effects of domestication facilitated the cropping of wheat, leading to an exponential increase in its economic importance [8].

As well as being a major source of carbohydrate in the form of starch, wheat seeds also provide a great source of protein in the human diet. Wheat endosperm proteins, the prolamins, can be divided into gliadins and glutenins according to their polymerization properties. They are the main components of gluten in wheat flour. The elasticity and extensibility of dough are the basic characteristics of breadmaking and are largely determined by the glutenin and gliadin content of the flour, respectively [9]. Indeed one of wheat’s main advantages is the unique ability of wheat doughs to be processed into different types of bread and other bakery products (including cakes and biscuits), pasta, and other processed foods [10]. The markets and the processing industry depend on different varieties of wheat with different quality attributes that meet the needs of specific products. Wheat is generally traded according to specific characteristics, namely, the grain protein content and hardness [11]. These market requirements have led to a more comprehensive and thoughtful approach to wheat breeding so as not to neglect essential protein content and protein quality [12,13]. 

The present work aims to describe the history of wheat with a particular focus on its singular characteristics making it almost ubiquitous in the human diet, the ability to form the gluten viscoelastic network. Worldwide production, market requirements, consumption habits, and research trends are also summarized in this work. 

## 2. Origin and Evolution

The history of wheat from about 10,000 B.C. is an important part of the history of agriculture [2]. Wheat is thought to have first been cultivated in the Fertile Crescent, an area in the Middle East spreading from Jordan, Palestine, and Lebanon to Syria, Turkey, Iraq, and Iran [14]. The earliest cultivated species are hulled (glumed) wheats and comprise all three polyploidy levels known in *Triticum* spp., diploid, tetraploid, and hexaploid [14]. These species are einkorn (*Triticum monococcum* L.), emmer (*Triticum Dicoccum*), and spelt (*Triticum spelta*), and their corresponding wild ancestors are still found in these territories [15,16]. However, the domestication of these three species caused considerable genetic erosion that has since been reinforced through modern breeding practices with the unwanted consequence of increased susceptibility or vulnerability to environmental stresses, pests, and diseases [8].

**Figure 1:**
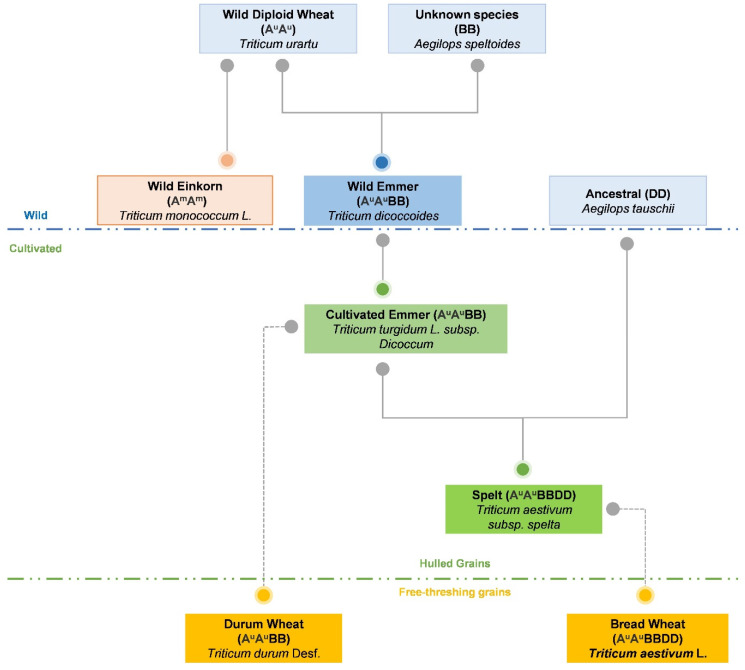
Phylogeny of the domesticated species of durum wheat *(Triticum durum* Desf.) and common wheat (*Triticum aestivum* L.).

Wheat belongs to the *Poaceae* family [17,18], which includes the *Triticeae* tribe, the division with the most economically important cereals. The *Triticeae* tribe includes 14 genera, grouped into the subtribes *Triticinae* and *Hordeinae*, and the production of amphiploids and interspecific hybrids suggests there is genetic or cytoplasmic compatibility between the different genera [19]. The most representative wheat species of this tribe are hexaploid *Triticum aestivum* L. (A^u^A^u^BBDD; 2n = 6x = 42), tetraploid *Triticum turgidum* L. (A^u^A^u^BB; 2n = 4x = 28), and diploid einkorn *Triticum monococcum* L. (A^m^A^m^; 2n = 2x = 14) [8]. Currently, the most economically important species are tetraploid durum wheat (*Triticum turgidum* subsp. *durum* (A^u^A^u^BB; 2n = 4x = 28) and hexaploidy bread wheat [8,11]. The origin of the latter two wheat species was the result of two polyploidization events (Figure 1).

The first of these occurred with the association of the genomes of two diploid species: one related to the wild species *Triticum urartu* (A^u^A^u^; 2n = 2x = 14) provided the A genome and the other yet unknown species from the *Sitopsis* provided the B genome. This produced the allotetraploid wild emmer wheat (*Triticum Dicoccum*; A^u^A^u^BB; 2n = 4x = 28) [20,21]. With the genetic resources of both ancestral diploids, this cultivated allotetraploid wheat is generally more vigorous, giving higher yields and adapting to a wider range of environmental conditions compared to its parents [22]. The second event occurred between *T. turgidum* and *Aegilops tauschii* (DD), a wild diploid species, which produced the allohexaploid early spelt (*Triticum aestivum subsp. spelta;* A^u^A^u^BBDD; 2n = 6x = 42) [8,20]. Initially, *T. aestivum* L. and *T. turgidum* produced sterile hybrids; however, a duplication of chromosomes in gametes or progeny gave rise to fertile species [23]. 

Initial cytogenetic studies of the origin of wheat proposed the diploid species *T. monococcum* L. as the donor of the A genome in the tetraploid species of both evolutionary lines [24]. However, recent studies have shown that variations in the repetitive nucleotide sequences in the A genome, present in both wheat tetraploid species, were more related to the *T. urartu* genome than to the *T. monococcum* L. genome [25,26]. The origin of the B genome remains unresolved. In polyploid wheat, the B genome constitutes the majority of the genomic component and as the genetic variation at the DNA level is high, the true donor of the B genome since polyploid formation has been very difficult to establish [26]. There are many strands of evidence (morphological, geographical, cytological, genetic, biochemical, and molecular) that suggest the section *Sitopsis* of *Aegilops*, namely *Aegilops speltoides* (genome S), as the B genome donor of both soft and hard wheat [26,27]. The ambiguous nature of the B genome remains a subject of study with several possible theories: (1) the parent is extinct; (2) the diploid parent still exists but has yet to be discovered; (3) in the formation of the allotetraploid, the diploid B genome donor has undergone several changes; and (4) in the condition of allopolyploidy, the B genome evolves rapidly due to several chromosomal structural changes, namely, due to the introgression of chromosomal segments of other allopolyploid or diploid species [27]. In contrast, the D genome is derived from the diploid progenitor *Aegilops tauschii* [16]. This genome carried genes and alleles that favored adaptation to the more continental climate of central Asia, thus allowing hexaploid wheat to be cultivated more widely than emmer wheat [28]. Khorasan wheat (*Triticum turgidum* ssp. *turanicum*) is also an important ancient species for the appearance of durum wheat, as it has morphological characteristics similar to those of durum wheat. There is no concrete data on its origin; however, through DNA analysis, it is likely that the species originated from a spontaneous cross between durum wheat and wild wheat [29].

In fact, wheat is a useful model organism in which to study the evolutionary theory of allopolyploid speciation, and consequently, the study of its adaptation to domestication [26]. The domestication of *T. aestivum* L. and *T. durum* Desf. began a selection process that increased the adaptation of these wheat species as well as their cultivation on a large scale [30]. This is attributable, in part, to the allopolyploid genome structures [20]. Allopolyploids contain two or more diverged homoeologous genomes, a fusion of different genomes [31]. Alloploidization accelerated the genome evolution of wheat through changes like gene loss, gene silencing, gene activation, and duplications [31,32]. Two evolutionary processes in particular were crucial in domestication [2]. A natural mutation at the *Q* locus of chromosome 5A modified the effects of recessive mutations at the *Tg* (*tenacious glume*) locus and thus made the wild emmer wheat and early spelt easier to thresh, later evolving into the free-threshing ears of durum wheat and common wheat, respectively, constituting a bridge between primitive wheat and modern wheat [8,14,16]. The fragility of the glumes of the two modern wheats is a technological advantage that facilitates the grinding of grain into flour, whereas the grains of more primitive species are covered by glumes (shells) that even after harvest make grinding difficult [14]. The other evolutionary process was the loss of ear fragmentation at maturity, resulting from mutations in loci, such as the *brittle rachis* (*Br*) gene, which allows the dispersion of seeds in natural populations [22,33]. The chromosomal location of *Br* in emmer wheat is on chromosomes 3A and 3B, but the equivalent in einkorn wheat is still unknown. In this regard, the selection of the non-breaking characteristic led to modern wheat varieties superseding einkorn, emmer, and spelt wheats [33]. 

## 3. Production

Despite modern wheat varieties (durum wheat and common wheat) having a higher yield potential, they have disadvantages compared to old varieties, particularly in terms of reduced tolerance of abiotic and biotic stresses, such as diseases, pests, drought, heat, cold, salinity, pollution, and shortage of soil nutrients [14]. The varieties we know today are the outcome of breeding programs that mainly selected and developed grains with specific characteristics, such as higher yield, superior breadmaking quality, efficient use of nutrients, and resistance to biotic and abiotic stresses. Selection is done by phenotyping and/or genotyping, using techniques such as sequencing or molecular markers [34,35,36]. 

In the 1960s, the world’s ability to deal with the increasing population and food demand was desperate (especially in developing countries), but this phenomenon stimulated a dramatic increase in cereal production in many of these countries, and large-scale hunger and the associated social and economic turmoil were averted [37]. Several genetic traits were selected to improve the yield, stability, and large-scale adaptability of rice, corn, and wheat [38]. The introduction of high-yielding wheat varieties was accompanied by massive application of chemical fertilizers and pesticides. The impact of these advances was so huge that it was called the Green Revolution [39]. 

The traditional and older varieties of wheat are tall and leafy, with very brittle stems. The Green Revolution brought greater productivity to wheat, mainly because of the introduction of cereal dwarfing genes [40]. The insertion of dwarfing genes from the Japanese variety ‘Norin 10’ made it possible to simultaneously increase the yield potential, harvest rate, responsiveness to fertilizers, and resistance to biotic and abiotic diseases [38,41]. In fact, more than 70% of commercial wheat varieties currently grown have the ‘Norin 10’ dwarfing genes in their genomes [39]. ‘Norin 10’ contains two dwarfing genes, *Rht1* and *Rht2*, which are semi-dominant alleles of homoeologous genes on chromosomes B and D, respectively [39]. The effect of each gene on plant height is similar and their combined effect is additive [42,43]. This comes from the fact that these gene products act as transcription factors in gibberellin signaling. Gibberellin is an essential endogenous regulator of plant growth [44] and the alleles that regulate dwarf genes not only reduce plant height, but also reduce responsiveness to gibberellin levels [45]. The dwarf genes also decrease the length of the coleoptile, which reduces the seedling survival rate and hence population density, a disadvantage of their use [46,47].

**Figure 2:**
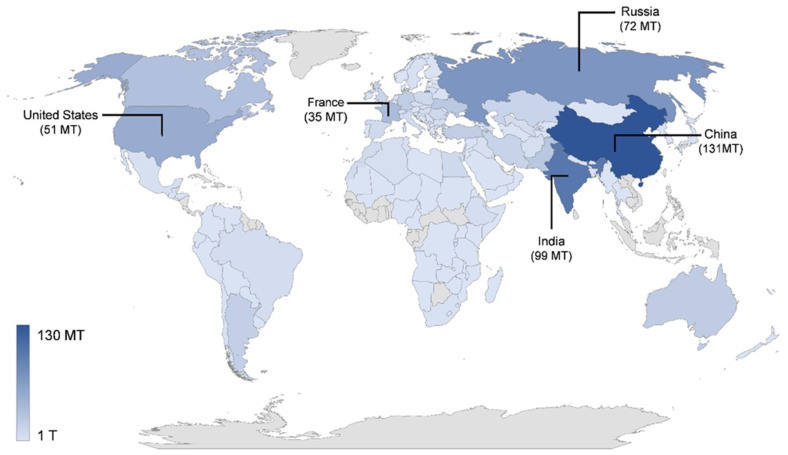
Worldwide wheat production. Data indicate the total wheat production in megaton (MT) in the year 2018 per country FAOSTAT [1].

Nowadays, about 95% of the wheat grown in the world is common wheat, used mainly as whole flour and refined flour to produce a wide variety of flat and fermented breads and for the manufacture of a wide variety of other bakery products. The remaining 5% is mostly durum wheat used to produce semolina (coarse flour), the main raw material for the manufacture of a wide variety of different baking product [6,11]. Figure 2 shows the production of wheat worldwide. The top five wheat producers are China, India, Russia, USA, and France [1]. 

**Figure 3:**
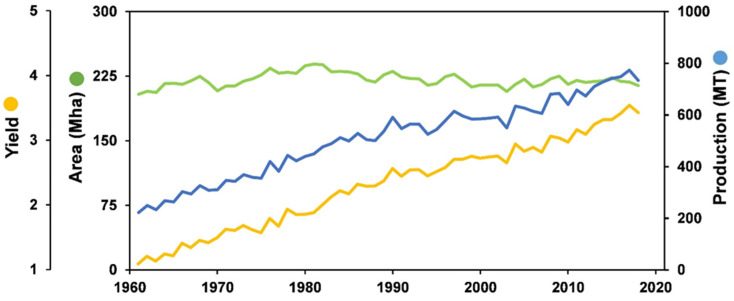
The world wheat production, harvested area, and yield between 1960 and 2018 FAOSTAT [1]. Through the indexmundi database, we were able to verify that China, India, and Russia had a positive production growth rate between the years 2018–2020 while, on the contrary, the USA had a decreased production growth rate [49].

The world’s wheat harvested area has remained relatively stable since 1960, but in the same period, production has risen significantly. Combining these data clearly shows that wheat production became much more efficient over the years, in part due to the introduction of hybrid crops that allowed an increase in yield (Figure 3) [1,48].

Sustainable food production requires environmentally friendly agricultural practices. Limiting the number of species or varieties of a species results in a huge loss of genetic diversity with associated negative impacts on the vulnerability of ecosystems and species extinction, and limiting the ability of farmers to respond to future agricultural needs [14]. The disadvantage of today’s high-yield high-protein wheat varieties is that they require large amounts of agrochemical input, such as fertilizers, pesticides, and herbicides. Additionally, climate change and global warming can have adverse effects on both the yield and quality of wheat. An alternative to overcome these obstacles is to use older varieties of wheat and landraces, like einkorn, emmer, and spelt, that have never been subjected to breeding practices [12,13]. 

## 4. Wheat Consumption Habits 

The consumption of bread, especially bread made with whole wheat flour and multigrain flour, is tending to increase in developed countries, especially due to the increased awareness of the need to reduce the consumption of simple carbohydrates, fats, and cholesterol and increase the consumption of complex carbohydrates, dietary fiber, and vegetable protein [50]. Each type of bread has its own characteristics, processing conditions, and specific requirements for the final product [11]. 

Durum wheat is widely used in regional foods (pasta, flat bread, couscous, and hamburgers) in Europe, North Africa, and Western Asia [10,16]. In Italy, durum wheat is traditionally used in the production of pasta, now consumed worldwide [51]. Durum wheat flour is used alone or mixed with other flours and is widely used in Mediterranean countries to make bread [10]. Couscous is one of the major food staples in North African countries, such as Egypt, Libya, Tunisia, Algeria, and Morocco [52]. Durum wheat has also been used in North America to make a ready-to-eat puffed breakfast cereal and in Germany, the noodles in kugel, a sweet pudding eaten as a dessert, are made of durum wheat [53]. 

Common wheat is generally milled into flour (refined and whole meal) and is used in breads (fermented, crushed, and steamed), pasta, cookies, and cakes [11]. Indeed, fermented breads are popular worldwide. The medium-hard to hard wheats that yield strong doughs are more suitable for producing rolls for hamburgers and hot dogs, for the semi-mechanized or manual production of typical French bread, as well as flat bread types like Arab baladi bread, Indian chapati, and Mexican tortilla [54,55]. The light dough made from soft wheat is suitable for Asian bread cooked with steam [56]. Waxy wheat has been developed with low levels of amylose or no amylose starch, which makes it possible to characterize the impact of starch on the quality and functionality of end-use food. Waxy wheat is often used as a source of blended flour to improve the shelf-life stability, processing quality, or palatability of baked and sheeted wheat products [57]. It has been shown that adding 40% waxy wheat flour to Chinese noodles improved the quality of dry white Chinese noodles and exhibited the shortest cooking time [58]. Another example of the use of waxy wheat is in the refrigeration and freezing of food products. This type of wheat has specific effects on gelatinization and retrogradation. To produce products for freezing purposes, waxy wheat is the best choice since it has a slow retrogradation rate [59]. 

The old wheat varieties are still being explored today as healthy alternatives to bread wheat [4]. Mainly for human consumption as different types of fermented bread, unleavened bread such as chapatti, and pancakes, old wheat varieties are also used as animal feed [4]. Ancient wheats have a nutritional composition that is different from modern varieties, such as resistant starch, carotenoids, phytochemicals, and antioxidants, that offer numerous health benefits [60]. These days, there is often a mixture of flours from these old wheats with modern varieties to make up for the lack of some components. For ex-ample, it has been observed that, compared to modern wheat, there is a reasonably high amount of protein in the emmer grain and a higher amount of lysine in the einkorn wheat [61]. Thus, baking bread with flours from these ancestral wheats can be more beneficial to health, as the high content of lysine and other health-promoting compounds in these wheats can complement those of modern wheat flours to achieve a better dietary balance [61,62]. Another reason why old wheat is important is that consumption trends are constantly changing, and there is an increasing demand for sustainable, regional, and artisanal products that have been manufactured in a way that conserves resources (promote biodiversity and reduce the ecological footprint) [63]. Old wheat products, such as breads made from Khorasan wheat, have good sensory properties and bread volumes almost as high as modern varieties. The old wheat species (einkorn, emmer, and spelt) are also used as whole grains for salads and used differently for processing, for example spelt used mainly for bakery products and the einkorn and emmer used mainly for pasta products [64,65]. These choices of using specific varieties to the manufacturing of specific products try to respond to the growing demand for a wider variety of products [65,66].

Bread made with emmer wheat flour is used worldwide, but especially in Switzerland. In Italy, it is used for *pane di farro* and to produce some pasta, but only in small quantities. In some rural areas of Italy and Iran, emmer is used as a source of carbohydrates in meals in the same way as rice [60]. 

Spelt flour and bread made with spelt have become increasingly popular in many countries due to the nutritional properties of this grain. Specifically, spelt bread has a higher protein content, a higher lipid content, a more desirable fatty acid profile, and higher percentages of several nutrients compared to common wheat [67]. From an agronomic point of view, compared to other wheats, spelt is more resistant to diseases and various pathogenic fungi, so it requires less fertilization, pesticides, and herbicides and, as the seeds are covered by the husk, no chemicals are needed before sowing [68,69]. With the increasing interest in organic farming, these characteristics have led to a higher global interest in the production of this species that may have several impacts on human nutrition, health, and sustainability [67]. On the other hand, general consumption habits are shifting to a gluten-free diet, as people are removing wheat from their diet without any medical advice and, consequently, are not consuming the necessary nutrients of wheat [70]. This demand for gluten-free products can arise from the belief that gluten-free and sugar-free foods help people overcome problems, such as bloating, indigestion, and others. Thus, bakery products that claim to be fat-free, sugar-free, gluten-free, whole wheat, and salt-free are popular with health-conscious consumers. According to the Agriculture and Horticulture Development Council, an average increase of 10% each year is expected for the next three years in the free-food market, including bakery products [71]. In the case of people diagnosed with disorders associated with gluten, such as celiac disease, some work has been carried out exploring the potentialities of sourdough bread. Basically, wheat is leavened using a long-time fermentation by sourdough, a cocktail of acidifying and proteolytic lactic acid bacteria, capable of hydrolyzing Pro-rich peptides, including the 33-mer peptide. Thus, a wheat product fermented with sourdough reduces its immunogenicity for celiac patients [72,73,74].

Considering the worldwide consumption of wheat products and the great impact of the growing world population, several techniques have been used to address these demands. One of the alternatives is the production of frozen dough products as they have a considerably longer shelf life. However, during freezing, there are structural changes induced in the gluten network [75]. Some studies indicate that these changes occur mainly in the α-helices, β-turns, and antiparallel-β-sheet structures, while the β-sheet content is not affected by the duration of frozen storage [76,77]. Other studies have led to the conclusion that changes in protein structure are essentially due to protein aggregation and, consequently, to gluten deterioration [78]. Thus, freezing, the frozen storage temperature, and temperature fluctuations during storage lead to a loss in dough and bread quality, resulting from lower yeast viability and bread volume and increased firmness of the bread crumb and mass weight loss [79]. To overcome this obstacle in freezing, different compounds are often added to improve the quality of the frozen dough. For example, the addition of NaI leads to an increase in the β-turns at the expense of intra- and intermolecular β-sheets. This promotes the increase in water–protein interactions, reducing protein–protein interactions, and, consequently, improving the quality of frozen dough [76,80]. 

## 5. Nutritional Value and Health Impact

Wheat is a good source of complex carbohydrates, with the starchy endosperm being the major storage tissue and accounting for about 80% of the wheat grain. Unlike simple carbohydrates, such as sugars, complex carbohydrates are preferred as they provide a more sustainable energy source as they gradually release energy according to the body’s needs [50]. Additionally, diets high in these complex carbohydrates lead to fewer health problems [81] and wheat starch provides a good source of energy in nutrition (1550 kJ/100 g) [50,82]. Compared to ancient wheats, the carbohydrate contents in durum (71%) and common wheat (75%) are slightly higher than, for example, spelt (68%) and einkorn (67%), which provide lower carbohydrate contents [65]. Fiber is also a very important dietary factor. The majority of dietary fiber in wheat is found in the white layers, and after wheat milling, the resulting white flour contains 2.0–2.5% fiber [83]. Thus, 40 g of white wheat bread can provide about 1 g of dietary fiber, whereas a similar serving of whole wheat bread can provide 3–4.5 g of dietary fiber. A high-fiber diet is positively associated with health benefits [84,85]. As well as the carbohydrate content, the ancient varieties, spelt (12%), einkorn (9.8%), and emmer (9.8%), have a lower fiber content than common wheat (13.4%) [13,65,86].

Lipids are a relatively minor component (2.5–3.3%) of wheat caryopsis, and 35–45% of wheat lipids are in the endosperm. Wheat has significant levels of lipids of various types, such as acylglycerols, fatty acids, and phospholipids, between others [87]. Lipids play an important role in the human diet as they are a high energy source and form the structure of cell membranes [88]. Although grain lipidomics is rather complex, there is experimental evidence that ancient wheats (einkorn, emmer, and spelt) have characteristic lipidomic profiles that are different from each other [89]. In general, ancient wheats, except for eikorn, have a higher lipid content than common wheat [90,91], but einkorn has higher monounsaturated fatty acids, lower polyunsaturated fatty acids, and lower saturated fatty acids than durum wheat, which has been proven to be good for human health [61]. 

**Figure 4:**
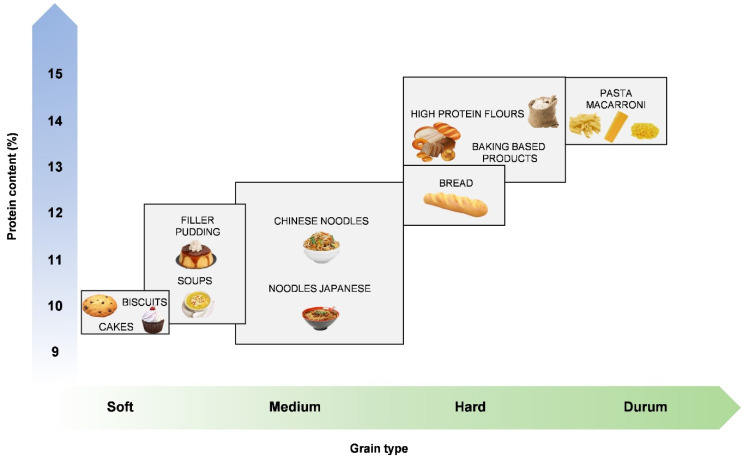
Examples of the most appropriated varieties according to the hardness of the grain and protein content for specific final products.

Wheat, depending on the wheat genetic background and fertilizer inputs, can provide a protein content. Nevertheless, durum wheats provide more protein than most other cereals and the distribution of essential amino acids is at least as good or better than other cereals [50]. The protein content of wheat can, in general, vary between 9% and 18% of the grain weight [16,92]. For example, common wheat intended for the production of cakes and/or biscuits will ideally have a protein content of 7–11%, while wheat intended for the production of high-volume loaf bread typically requires a 12% or higher protein content (Figure 4) [83]. When compared to ancient wheats, modern wheat has a possibly lower protein content, as the grain of modern wheat is larger and heavier, which produces a larger starchy endosperm, which, in turn, lowers its protein content. In fact, in einkorn, the protein content is 15.5–22.8% and the percentage of amylose is 23.8%, whereas in common wheat, the protein content and amylose percentages are 12.9–19.9% and 28.4%, respectively [14]. 

Furthermore, the amino acid composition of wheat lacks essential amino acids, such as lysine, threonine, and methionine [48], exhibiting a higher content of glutamine (>30%) and proline (>10%) residues. For example, glutamine and glutamic acid are involved in several important biological processes, such as gene expression regulation, intracellular protein turnover, nutrient metabolism, and oxidative defense [93]. On the other hand, proline residues confer some resistance to gluten proteins to proteolysis by digestive enzymes in the gastrointestinal tract [94]. As gluten is rich in proline, it produces a compact and hard structure that usually presents difficulties in digestion and elimination [95]. Some of these digestion-resistant peptides are believed to be related to adverse immune processes in susceptible people [96]. In celiac disease, some of these peptides are responsible for the onset of the disease, triggering an abnormal immune system response in genetically predisposed individuals [97,98]. There is evidence that the D genome of bread wheat has more immunogenic epitopes than the A and B genomes. Thus, it is expected that diploid and tetraploid wheats (*T. monococcum* and *Triticum durum*, for example) tend to contain less immunogenic epitopes for celiac patients than bread wheat [99]. In general, gliadins are considered the main cause of problems for celiac patients and the most immunogenic sequences occur in the N-terminal repetitive domain of α-/β-gliadins, which mainly consist of glutamine, proline, and aromatic amino acids [72,100]. Currently, several studies have explored the immunotoxicity for celiac patients of ancient and modern common wheat varieties and also other wheat species [101]. A study focused on the content of celiac-related epitopes of ancient and modern *T. aestivum* and *T. durum* wheat varieties, and also spelt showed the following relationship: spelt > wheat landraces > modern wheat varieties = tetraploid varieties [102]. It was hypothesized that the breeding programs could have led to the increase of the glutenin content in wheat, therefore decreasing the gliadin to glutenin ratio [102]. In the same way, Prandi et al., analyzing the gluten peptides released after in vitro digestion of different old and modern *Triticum* varieties, found that older varieties compared to modern varieties had higher amounts of immunogenic peptides, thus concluding that both ancient and modern varieties are not safe for consumption in the case of celiac patients [103]. Furthermore, other authors concluded that the total quantitative amount of immunogenic peptides is not directly related to the ploidy level or their origin (ancient/ modern). For example, common wheat (hexaploid) showed the same amount of immunogenic peptides as emmer and durum wheat (tetraploids) [103,104]. Nevertheless, all the results point to a natural inter-varietal genetic difference affecting the amount of celiac-related epitopes, which makes it possible to identify accessions presenting a lower amount of T cell stimulatory epitope sequences and therefore are more suitable for wheat breeding programs [101,105].

In addition to the main components of wheat, proteins, carbohydrates, and lipids, wheat grain is also an important source of phytochemicals, vitamins, antioxidants, and macro and micronutrients that are important components of human health [83]. 

## 6. Functional and Technological Properties 

### 6.1. Grain Hardness

The hardness of the wheat grain refers to its resistance to milling. The harder the wheat, the greater its resistance and the more difficult it is to grind into flour [83]. Between common wheat and durum wheat varieties, there are significant differences in the composition of the grain and in the quality of wheat processing, i.e., a variety may be suitable for the production of certain foods but unsuitable for the processing of others [11,50]. For example, as summarized in Figure 4, for a product like leavened bread, medium to hard grains with 11% to 13% of protein are normally used, while for cookies, cakes, and Chinese noodles, soft to medium grains with 8% to 12% protein are more suitable [11].

The discovery of the starch granule protein, friabilin, which influences the texture and quality of the wheat grain, provided a biochemical basis for evaluating grain texture. The friabilin protein complex regulates the degree of adhesion of starch granules to the protein matrix, a factor of great importance in determining grain hardness [106]. The variation in grain texture (hardness or softness) is a characteristic inherited and controlled by a single locus referred to as *Hardness* (*Ha*), which comprises three genes: *Pina* codes for puroindoline a, *Pinb* codes for puroindoline b, and *Gsp*-1 codes for a less abundant protein called grain softness protein [28]. 

In wheat grain, the accumulation of friabilin is dependent on the softness/hardness genes *Ha*/*ha* [106]. Soft wheats have a wild-type form (*Ha*) whereas hard wheats have a recessive (*ha*) or mutated form [107]. Additionally, variation in *Pin* genes significantly affects the grinding characteristics and quality of the final product, for example, the wild-type *Pina* and *Pinb* genotype results in a soft-textured phenotype, while mutation in one or both genes results in a more rigid phenotype [108]. Durum wheat represents a generally tougher class of wheat, but the relationship to *Ha/ha* is still unclear [106].

The texture of the endosperm is also a crucial factor for wheat producers as this characteristic influences certain physical properties, such as the particle size and density of the flour, risk of damaging starch, water absorption, and milling yield [109]. The cell contents of common wheat endosperm fracture, leaving the starch granules intact, and resulting in a wide particle size distribution. The main physical difference between hard and soft wheat endosperm is that the starch granules of soft wheat are surrounded by a protein matrix. On the other hand, in hard grains, crushing and grinding the grain is more difficult and produces flour with a coarse texture because more of the starch is damaged. In turn, endosperms of harder wheats have a greater capacity to absorb water and, consequently, are more easily hydrolyzed by alpha-amylase [107]. 

It is difficult to reduce the particle size of durum wheat flour since the grain tends to fragment along the lines of the cell boundaries, and on average, particles tend to be relatively uniform and larger than particles from soft wheat flours [110]. 

### 6.2. Wheat Proteins

#### 6.2.1. Non-Gluten Proteins

Wheat proteins can be divided into two groups: gluten proteins and non-gluten proteins. The group of non-gluten proteins is divided into albumins (soluble in water) and globulins (soluble in neutral saline solutions) and play mainly structural and metabolic functions and a minor role in wheat quality [111]. For example, albumins and globulins include enzymes and enzyme inhibitors that regulate development at different stages in wheat growth [112]. These proteins are also involved in IgE-mediated food allergies [113,114]. Albumins are soluble in water and globulins are soluble in neutral saline solutions. Non-gluten proteins are mostly monomeric; however, albumins and globulins tend to form polymers through the formation of inter-chain disulphide bonds [115]. Tomic et al. developed a study in which they focused on finding the relationship between particular albumin fractions and the enzymatic activity of wheat flour and the rheological properties of the dough [116]. The study included albumins like α-amylase, α-amylase/protease inhibitors (13 and 16 kDa), as well as enzymes with different physiological functions (62 kDa). This study showed that the 15–30 kDa albumin fraction was shown to influence the rheological properties of the dough, especially water absorption and resistance to extension. With regard to the 5–15 kDa, 30–50 kDa, and 30–60 kDa albumin fractions and the proteolytic and α-amylolytic activity, no relationship was found regarding the enzymatic state of the flour [116]. 

Recently, other wheat proteins, such as ALP (avenin-like protein), have been associated with wheat processing properties and bread-making quality [117]. ALP-coding genes were mapped at the long arm of chromosome 4A and at the short arms of chromosomes 7A and 7D in bread wheat. The ALP proteins, in addition to improving the dough mixing properties, also possess antifungal functions, showing an important potential for wheat breeding [118]. On the other hand, a novel gene involved in breadmaking properties was reported. The wbm (wheat bread making) gene was demonstrated to have a significant effect on gluten quality, gluten strength, gluten extensibility, and bakery quality [119].

#### 6.2.2. Gluten

Gluten, one of the first protein components to be chemically analyzed, was first described by Giacomo Beccari in 1728 [16]. Beccari reported preparing a water-insoluble fraction of wheat flour that he called “glutinis” [120]. The gluten was later defined as being composed of prolamins and glutelins by Thomas Burr Osborne between the years 1886 and 1928 based on his study of plant proteins. It was Osborne who classified proteins into groups based on their solubility [121]. For a long time, based on Osborne’s definition, gliadins were thought to be the prolamins and distinctly different from glutenins thought to be glutelins. However, biochemical and molecular studies, corroborated by genetic studies, have proven that all gluten proteins are structurally and evolutionarily related and can be collectively defined as prolamins [122]. According to Osborne, the gliadin and glutenin together represent up to 85% of the total protein content within wheat endosperm (gliadins ~40%, high- and low-molecular-weight glutenin sub-units ~10% and ~30%, respectively) [123]. 

Gliadins are mostly monomeric proteins with a molecular weight that ranges from 28 to 55 kDa. They are separated into four groups on the basis of mobility at low pH in gel electrophoresis: α-, β-, γ-, and ω-gliadins [124]. α-, β-, and γ-gliadins are rich in cysteine and methionine residues through which intramolecular disulphide bonds form, imposing a specific conformation on the polypeptide [125]. By contrast, ω-gliadins usually do not have any cysteine residues. An exception is a group of mutant ω-gliadin forms that have a single cysteine residue, which allows them to establish intermolecular disulphide bonds and to be incorporated into the glutenin polymer [126]. Based on analysis of the complete or partial amino acid sequence, amino acid composition, and molecular weight, gliadins can be redivided into four different types: ω5-, ω1,2-, α/β-, and γ-gliadins. The α-, β-, and some γ-gliadins are also encoded by the short arms of chromosome 6 (*Gli-2*) whereas the majority of γ- and ω-gliadins are encoded by the *Gli-1* genes on the short arms of chromosome 1 [127]. Specifically, they are located on chromosomes 1A, 1B, and 1D at the three homeologous *Gli-A1*, *Gli-B1*, and *Gli-D1* loci and on chromosomes 6A, 6B, and 6D at the *Gli-A2, Gli-B2*, and *Gli-D2* loci [128]. 

Glutenins are polymeric proteins with a molecular weight that can exceed 34,000 kDa [129]. Based on their mobility when separated through polyacrylamide gels, they were classified into high-molecular-weight glutenins (HMW-GSs; 70–90 kDa) and low-molecular-weight glutenins (LMW-GSs; 30–45 kDa) [129]. The genes encoding HMW glutenins are located on the long arm of chromosomes 1A, 1B, and 1D at the *Glu-A1*, *Glu-B1*, and *Glu-D1* loci, respectively [130]. The *Glu-A1* locus can present more than 21 allelic variants in *T. durum* Desf. In *T. aestivum* L., the *Glu-B1* locus has more than 69 alleles and the *Glu-D1* locus more than 29 [131]. In each common wheat variety, three to five different HMW-GSs can be found. *Glu-A1* encodes one subunit, *Glu-B1* encodes one or two subunits, and *Glu-D1* encodes two subunits. These subunits can be further classified into x-type and y-type. In particular, y-type subunits do not have a particularly positive effect on dough consistency. On the other hand, x-type subunits are responsible for the formation of high-molecular-weight protein aggregates resulting from their ability to form linear polymers through cysteine residues [128,132,133]. 

According to molecular weight and composition, LMW-GSs can be divided into groups B, C, and D [134]; and LMW-GS B type glutenin divided into three further categories according to the first amino acid in the peptide chain, LMW-m (methionine), LMW-s (serine), and LMW-i (isoleucine) [134,135]. The genes that encode them are on the short arms of chromosomes 1A, 1B, and 1D at the *Glu-A3*, *Glu-B3*, and *Glu-D3 loci*, respectively [130].

### 6.3. Effect of Gluten Proteins on Wheat Functionality

Gluten accounts for about 85% of the protein component of wheat endosperm [136]. The gluten matrix and the functions it performs determine the end-use quality of wheat [137]. The viscosity and extensibility of the dough is mainly related to the properties of hydrated gliadins, while the strength and elasticity of the dough comes essentially from the cohesive properties of hydrated glutenins [138]. Furthermore, the gluten protein fractions contain many individual components with much allelic variation between varieties. For example, a typical wheat variety can have up to 60 different gluten proteins [139]. 

In 1981, Payne and colleagues demonstrated that HMW-GS variation influences the breadmaking quality of wheat flour. They correlated the presence of two HMW-GS with breadmaking quality. Subunit 1 expressed from the *Glu-A1* locus and subunits 5 + 10 expressed from the *Glu-D1* locus were related to good quality, when compared to null and 2 + 12 sub-units, respectively [140]. Years later, in 1987, when analyzing 84 varieties of British-grown wheat, they developed a scoring system (based on the SDS-sedimentation test) to assess the individual contribution of each subunit expressed from the *Glu-1* locus to the flour quality. The score for a variety could be obtained by adding the points attributed to each individual subunit [141]. The relative contribution of each HMW-GS locus to the characteristics of the flour is *Glu-D1* > *Glu-B1* > *Glu-A1*. The protein content is independent of this contribution ratio, but the contribution of each locus depends on the expressed subunits and wheat variety in question [135,142].

The effects of gliadins and LMW-GSs on the characteristics of flour are not yet fully understood as the genetic link is not fully established and it is difficult to distinguish these proteins in polyacrylamide gels [143]. For example, the genes encoding the monomeric prolamins are strongly linked to the genes of the LMW glutenins at the *Glu-3* locus and the genes that encode gliadins and LMW glutenins (type C and D) are interspersed within the *Gli-1* loci [143,144]. However, there is evidence that allelic variation between the LMW glutenin subunits has an influence on the breadmaking quality, with the alleles of the *Glu-D3 locus* having a lesser influence on the quality characteristics than the alleles of *Glu-A3* and *Glu-B3*, even though the *Glu-D3 locus* is larger than the others [145,146]. 

The effect of specific LMW-GSs on gluten strength has been determined in durum wheat. Glutenin subunit LMW-2 and its variants confers stronger gluten characteristics than LMW-1 subunits. There are still many unknowns about the relationship between LMW-GS composition in bread wheat and gluten strength. This is partly due to the larger number of LMW-GSs in bread wheat than in durum wheat (the D genome is not present in durum wheat) [11].

In addition to the individual effects of gliadins and glutenins, the gluten network cannot be formed without the presence of these two proteins, and the ratio between glutenins and gliadins is an essential quality determinant for its end use [147]. Studies revealed inter-varietal variation in the Gli/Glu ratio. Nevertheless, whereas common wheat is typically 1.5–3.1, that of ancient wheats was much higher (spelt: 2.8–4.0; emmer: 3.6–6.7; einkorn: 4.2–12.0) [148]. This ratio is fundamental for the breadmaking quality, such as the high volume of bread. When the content of gliadins is higher than that of glutenins, the mixing time is shortened and the stability of the dough reduced, while a greater amount of glutenins, namely HMW-GS and LMW-GS, is positively correlated with bread-making quality [13]. Rodríguez-Quijano et al., in one of their studies about common wheat and spelt, demonstrated that in rheological terms, the Gli/Glu ratio was positively associated with the viscosity and extensibility of the dough and negatively associated with the strength properties so important for breadmaking [13]. Another study conducted by Dhaka et al. demonstrated that the Gli/Glu ratio showed a significant negative relationship with specific bread volume (r = −0.73), dough development time (r = −0.73), and stability of mass (r = −0.79) and a positive relationship with LMW-GS quantity (r = 0.72) [9]. Barak et al. came to identical conclusions, where higher Gli/Glu ratios are negatively associated with general bread quality parameters. 

### 6.4. Models of Gluten Structure and Function in Dough 

The strength of the dough, its extensibility, and resistance to kneading are rheological properties that are key parameters in baking [149]. Using small-strain dynamic rheology, a lot of research has been done to define the theoretical basis for gluten structure, relating it to the rheological behavior of dough or gluten [83].

#### 6.4.1. Pom-Pom Model 

Proposed by MacLeish and Larson, the pom-pom model addresses the rheological behavior of HMW branched polymer melts and gluten [150]. It is thought that these polymers are a relatively flexible HMW backbone with several branches, the pom-poms, that protrude from each end of the backbone. Therefore, the interaction between the branches and the surrounding polymers creates entanglements and the stretching of the backbone between those entanglements leads to strain hardening. The predictions of this model have also shown that strain hardening is affected not only by the number of branches, but also the distance between the entanglements [149].

#### 6.4.2. Loop-Train Model 

The loop-train model was proposed by Belton in order to explain the elastic properties of gluten, emphasizing the role of HMW-GS [151]. According to this model, interchain hydrogen bonds (trains) in gluten hold some regions of the protein chain while some unbonded regions form loops. When gluten is stretched, it causes the loops to extend so that protein chains slide over one another. The reestablishment of the loop-train equilibrium of the unstretched protein creates the elastic restoring force. One of the major critiques of this model is the fact that it has yet to be applied to three-dimensional systems, and that HMW-GS would influence gluten behavior by affecting the properties and size of the gluten network instead of acting as distinct units [83].

#### 6.4.3. Particle-Gel Model

Hamer and co-workers [152,153] proposed a model of glutenin in dough that is based on large aggregates of glutenin. These authors believe that the gluten macropolymer (GMP), the highest molecular weight and consequently least soluble glutenin fraction, affects dough behavior. The idea is that the GMP forms a gel (or particle network) that influences the dough’s viscoelastic properties [83]. 

#### 6.4.4. Linear Glutenin Hypothesis

This hypothesis is a model based on polymer science and is built around the concept of individual chain unfolding within assemblies of glutenin chains when under stress [154,155]. It is proposed the linear branches are composed of dimers of chain-extending LMW-GSs and dimers of chain-extending and chain-terminating LMW-GSs (predicted to be the final subunits in the structure). These dimers come from the head-to-tail arrangement of chain-extending LMW-GSs [156]. As a consequence, elastic properties come from the tendency of those chains to refold to the lowest free energy states. This hypothesis is consistent with the dominant linear molecular structure of glutenin proven by Ewart [157].

Although some models have achieved widespread acceptance, the structure of gluten and the relationship with its rheological properties still need to be further clarified [83,149].

## 7. Conclusions

Wheat is one of the most important grain crops in the world. The domestication of the ancient varieties of einkorn, emmer, and spelt was the basis for the appearance of durum and common wheat. This domestication increased the yield of wheat through genetic changes that affected characteristics, such as brittle rachis, tenacious glumes, and height. Modern varieties have proved to be highly productive with superior quality concerning end-use.

Protein content is the main factor determining the nutritional and technical quality of wheat, and the quantity and composition of wheat protein are good indicators of the quality of the final product. The gluten viscoelastic network is a fundamental property to produce fermented bread and other foods.

Given the importance of wheat, the continued study of this cereal on all fronts is essential. Plant species like wheat that are essential to human nutrition must evolve and adapt to the changing world climate. Disease, heat, and drought resistance are being addressed in current research. On this note, the demand for diversified, nutritious, and healthy wheat foods has led to a growing interest in nutrition research in ancient wheat, such as einkorn and spelt. These ancient wheats are a valuable source for improvement in opposition to modern wheat, which was already the subject of numerous alterations. Finally, although gluten-free habits have gained some adherence in recent years, often conditioned by some misconceptions associated with gluten, it should be noted that after 10,000 years, wheat is still one of the most representative foods in the human diet.

## Data Availability

Not applicable.
